# Lack of Evidence for a Direct Interaction of Progranulin and Tumor Necrosis Factor Receptor-1 and Tumor Necrosis Factor Receptor-2 From Cellular Binding Studies

**DOI:** 10.3389/fimmu.2018.00793

**Published:** 2018-04-23

**Authors:** Isabell Lang, Simone Füllsack, Harald Wajant

**Affiliations:** Division of Molecular Internal Medicine, Department of Internal Medicine II, University Hospital of Würzburg, Würzburg, Germany

**Keywords:** binding studies, *Gaussia princeps* luciferase fusion protein, progranulin, tumor necrosis factor, tumor necrosis factor receptor-1, tumor necrosis factor receptor-2

## Abstract

Progranulin (PGRN) is a secreted anti-inflammatory protein which can be processed by neutrophil proteases to various granulins. It has been reported that at least a significant portion of the anti-inflammatory effects of PGRN is due to direct high affinity binding to tumor necrosis factor receptor-1 (TNFR1) and TNFR2 and inhibition of tumor necrosis factor (TNF)-induced TNFR1/2 signaling. Two studies failed to reproduce the interaction of TNFR1 and TNFR2 with PGRN, but follow up reports speculated that this was due to varying experimental circumstances and/or the use of PGRN from different sources. However, even under consideration of these speculations, there is still a striking discrepancy in the literature between the concentrations of PGRN needed to inhibit TNF signaling and the concentrations required to block TNF binding to TNFR1 and TNFR2. While signaling events induced by 0.2–2 nM of TNF have been efficiently inhibited by low, near to equimolar concentrations (0.5–2.5 nM) of PGRN in various studies, the reported inhibitory effects of PGRN on TNF-binding to TNFR1/2 required a huge excess of PGRN (100–1,000-fold). Therefore, we investigated the effect of PGRN on TNF binding to TNFR1 and TNFR2 in highly sensitive cellular binding studies. Unlabeled TNF inhibited >95% of the specific binding of a *Gaussia princeps* luciferase (GpL) fusion protein of TNF to TNFR1 and TNFR2 and blocked binding of soluble GpL fusion proteins of TNFR1 and TNFR2 to membrane TNF expressing cells to >95%, too. Purified PGRN, however, showed in both assays no effect on TNF–TNFR1/2 interaction even when applied in huge excess. To rule out that tags and purification- or storage-related effects compromise the potential ability of PGRN to bind TNF receptors, we directly co-expressed PGRN, and as control TNF, in TNFR1- and TNFR2-expressing cells and looked for binding of GpL-TNF. While expression of TNF strongly inhibited binding of GpL-TNF to TNFR1/2, co-expression of PGRN had not effect on the ability of the TNFR1/2-expressing cells to bind TNF.

## Introduction

Tumor necrosis factor-alpha (TNF) is a pleiotropic cytokine which has not only crucially implicated in a variety of immunoregulatory processes in innate and adaptive immunity, but has also manifold roles in the control of tissue homeostasis (1). TNF is initially expressed as a trimeric type II transmembrane protein (memTNF) from which a soluble trimeric molecule (sTNF) is released by cleavage by the protease TNF converting enzyme (TACE) (1). Both sTNF and memTNF bind with high affinity to two types of receptors, tumor necrosis factor receptor-1 (TNFR1) and TNF receptor-2 (TNFR2). While memTNF binding results in strong activation of both TNFR1 and TNFR2, sTNF binding triggers efficient TNFR1 signaling but has no or only a modest effect on TNFR2 activity (1). TNFR1 and TNFR2 interact furthermore with high affinity with lymphotoxin-α (LTα), also named TNFβ, a soluble ligand trimer which is structurally closely related to TNF (1). TNFR1 and TNFR2 share a similar extracellular domain architecture comprising four cysteine-rich domains (CRDs) defining their affiliation to the TNF receptor superfamily (TNFRSF) (2). The crystallographic structures of TNF in complex with the ectodomain of TNFR2 and of LTα in complex with the ectodomain of TNFR1 have been solved. Both structures show that three molecules of TNFR1 or TNFR2 bind into the three grooves formed by three protomers of a ligand trimer (3, 4). Since the two TNF receptors have different types of intracellular domains with basically different binding partners, TNFR1 and TNFR2 elicit significantly different cellular responses upon activation. Excessive and/or chronic TNF activity has a pivotal role in various immune diseases and can contribute to various aspects of cancer development. TNF and its receptors are, therefore, considered as promising targets in a variety of diseases. Indeed, TNF inhibitors are already in clinically practice, since almost two decades and are powerful drugs in the therapy of Crohn’s disease, ulcerative colitis, psoriasis, and several arthritic diseases, including rheumatoid arthritis, psoriatic arthritis, and juvenile idiopathic arthritis. Although, five TNF-neutralizing biologicals have been approved, there are still enormous preclinical and clinical efforts to develop new drugs (antibodies, ligand mutants, small-molecules) inhibiting TNF, TNFR1, or TNFR2.

Progranulin (PGRN) or granulin precursor (Gene ID GRN) is a phylogenetically conserved unique protein without stringent homology to other proteins (5, 6). PGRN is a secretory glycoprotein expressed by a variety of cell types and present in blood and cerebrospinal fluid (5, 6). PGRN is composed and characterized by cysteine-rich so-called granulin domains. Human PGRN comprises N-terminal a truncated version of this domain type which is followed by seven complete granulin domains. PGRN can be proteolytically processed in most of the linker regions connecting the granulin domains resulting in the release of various granulin peptides covering one or more granulin domains (5, 6). Both PGRN and PGRN-derived peptides display complex biological activities, including stimulation of cellular proliferation, immune regulation, modulation of synaptic activity and neurogenesis (5, 6). In accordance with the latter, mutations in the GRN gene have been identified as cause of a familiar form of the neurodegenerative disease frontotemporal lobar degeneration (5, 6).

There is growing *in vitro* and animal model evidence that the immune modulatory effects of PGRN are based, at least in part, on the modulation of TNF signaling (7–19). The basis of this crosstalk, however, appears to be complex, since inhibitory and stimulatory effects of PGRN on TNF signaling have been reported. Tang et al. noteworthily identified PGRN as a TNF antagonist and reported, based on cell-free binding studies, high-affinities of PGRN for TNFR1 and TNFR2 which even exceeded those of TNF (7). This could explain the inhibitory effects of PGRN on TNF signaling. Two other groups, however, failed to demonstrate direct inhibition of TNF–TNF receptor interaction (20, 21). A third group reported that PGRN is unable to inhibit TNF-induced cell death (22). The researchers identifying PGRN as a competitive inhibitor of TNF binding suggested that these contradictory findings could result from different chip types used to analyze the PGRN–TNFR interaction in cell-free assays by help of the surface plasmon resonance (SPR) method and/or the use of PGRN from distinct sources/suppliers or different quality [Table 1 and Ref. (23, 24)]. Nevertheless, even under consideration of these unverified speculations, the available literature is still inconsistent with respect to the PGRN concentrations reported to inhibit TNF binding and TNF signaling (Table 2). The inhibitory effects of PGRN on TNF signaling in cellular assays have been observed at low, roughly equimolar nanomolar concentrations of sTNF and PGRN. In contrast, the demonstration of the inhibitory effect of PGRN on TNF-binding to cell expressed TNFR1/2 required a huge excess of 2–3 orders of magnitude of PGRN (7, 10, 14). The use of cell-free systems as well as the use of tagged and/or purified proteins can lead to misleading results in binding studies. We, therefore, performed cell-based competitive binding studies with TNFR1- and TNFR2-expressing cells and various PGRN variants, including non-tagged non-purified and thus maximally authentic PGRN derived from human embryonal kidney cells 293 (HEK293) cells. From these experiments, we gained no evidence for inhibitory effects of high concentrations (20–500 nM) of PGRN on the interaction of TNF with TNFR1 and TNFR2.

**Table 1:** Progranulin (PGRN) variants used to study the PGRN-tumor necrosis factor (TNF) crosstalk.

Variant and purification	Effect	Reference
PGRN-myc-6xHis, Ni-NTA purified	Inhibition of TNF signaling and TNF–TNFR1/2 interaction	(7)
PGRN-myc-6xHis, Ni-NTA purified	Inhibition of TNF-induced chemokine production	(11)
PGRN-myc-6xHis, Ni-NTA purified	Inhibition of TNF-induced chemokine production	(10)
PGRN-myc-6xHis, Ni-NTA purified	Inhibition of TNF-induced chemokine production	(12)
PGRN-myc-6xHis, Ni-NTA purified	Inhibition of TNF binding to Jurkat cells	(23)
PGRN-6xHis, purified (R&D Systems)	Inhibition of TNF signaling and TNF–TNF receptor interaction	(14)
PGRN-6xHis, purified (R&D Systems)	No effect on TNF signaling and TNF–TNFR1/2 interaction	(20)
PGRN-6xHis, purified (Sino Biologicals)	Anti-TNFR2 blocks PGRN-induced Akt signaling	(15)
PGRN-6xHis, purified (Sino Biologicals)	Neutralizing anti-TNFR2 blocks PGRN-induced signaling	(18)
mPGRN-6xHis, purified (R&D Systems)	Inhibition of TNF-induced osteoclastogenesis	(17)
PGRN-myc-6xHis	Inhibition of TNF-triggered ICAM1/VCAM1 induction	(13)
PGRN, untagged purified (Adipogen)	Enhancement of TNF-induced proliferation of Tregs	(9)
PGRN, untagged purified (Adipogen)	Inhibition of TNF-induced cytotoxicity	(19)
PGRN, untagged purified (Adipogen)	TNFR1 and TNFR2 binding in surface plasmon resonance	(23)
PGRN, untagged purified (Adipogen) mPGRN, untagged purified (Adipogen)	No effect on TNF signaling and TNF–TNFR1/2 interaction	(20)
mPGRN-6xHis, purified (R&D Systems) PGRN, purified (five prime therapeutics) N-TAP-PGRN,^a^ Strep-Tactin purified PGRN-C-TAP,^b^ Strep-Tactin purified PGRN-3xFlag, anti-Flag purified mPGRN-Fc, protein A purified	No effect on TNF signaling and TNF–TNFR1/2 interaction, all PGRN variants tested for their capacity to induce pERK in H4 glioma cells	(21)

*Inhibitory effects of PGRN on TNF-induced signaling or TNF–tumor necrosis factor receptor (TNFR)1/2 interaction are shown with white background, lack of effect(s) of PGRN on TNF signaling/TNF receptor binding are shaded in blue, and studies indicating that PGRN effects are mediated by TNFR2 activation are shaded in red*.

*^a^N-TAP, tandem Strep-II tag followed by the V5 epitope*.

*^b^C-TAP, tandem Strep-II tag followed by the Flag epitope*.

**Table 2:** Inhibitory effects of progranulin (PGRN) on tumor necrosis factor (TNF) activity and TNF receptor binding of TNF in intact cells.

TNF activity or binding assay	TNF conc. (ng/ml)^a^	PGRN conc. (ng/ml)^b^	Effect	Reference
NFκB signaling	10	225	Complete inhibition	Figures 6A,C of ref. (7)
NFκB reporter	10	9, 45, 225	IC50: approximately 45 ng/ml	Figure 6E of ref. (7)
NFκB regulated genes	10	225	Approximately 90% inhibition	Figure 6F of ref. (7)
p38/JNK activation	10	225	Complete inhibition	Figure 6G of ref. (7)
TNF inhibition of Treg activity	50	10, 50, 250	IC50: approximately 10 ng/ml	Figure S3A of ref. (7)
TNF toxicity	0.08	0–90	IC50: approximately 0.09 ng/ml	Figure S14D of ref. (7)
Treg proliferation	50	2, 20, 200	No inhibitory effect of PGRN, but enhancement at 2 and 20 ng/ml	Figure 1 of ref. (9)
Treg proliferation	20	2, 20, 200	No inhibitory effect of PGRN, but enhancement at 2 and 20 ng/ml	Figure 4 of ref. (9)
Gene induction	20	500, 2,500	80% to complete inhibition at 2,500 ng/ml	Figure 1 of ref. (10)
Gene induction	20	200	Approximately 50% to near complete inhibition	Figures 2 and 3 of ref. (11)
Gene induction	10	200	Approximately 50–90% inhibition	(12)
Gene induction	5	10, 50, 100	Approximately 50% inhibition with 100 ng/ml	(13)
Migration	100	250	Approximately 30%	(14)
Inhibition of osteoclastogenesis	10	5, 50	Strong inhibition	(17)
Cell death	0.1	250	Strong inhibition	(19)
Fluorescence-activated cell sorting (FACS)	250	75,000^c^ 375,000^c^	Reduction of mean fluorescence intensity approximately 30% Reduction of mean fluorescence intensity approximately 90%	Figure 1D of ref. (7)
FACS	Not indicated	5,000^d^	Reduction of mean fluorescence intensity approximately 30%	Figure 1B of ref. (23)
		25,000^d^	Reduction of mean fluorescence intensity approximately 90%	
		50,000^d^	Reduction of mean fluorescence intensity approximately 95%	
FACS	250	25,000^e^	Reduction of mean fluorescence intensity, quantification not possible due to missing indication of background staining	Figure 1 of ref. (10)
^125^I-TNF cell binding	0.05	0–250	Reduction of bound ^125^I-TNF approximately 50% with 250 ng/ml	(14)

*Inhibitory effects of PGRN on TNF-induced cellular responses are shown with white background, inhibitory effects on TNF–TNFR1/2 interaction are shaded in blue*.

*^a^TNF (MW 50,000) concentrations were converted as follows: 1 nM = 50 ng/ml*.

*^b^PGRN (MW 90,000) concentrations were converted as follows: 1 nM = 90 ng/ml*.

*^c^In a volume not indicated in the manuscript, cells were preincubated with 15,000 or 75,000 ng PGRN followed by addition of 50 ng biotinylated TNF. Finally, cell-bound TNF was detected using avidin-FITC in 200 µl. Indicated concentrations are based on the assumption that the latter volume has also been used in all other incubation steps*.

*^d^In a volume not indicated in the manuscript, cells were preincubated with 1000, 5,000 or 10,000 ng PGRN followed by addition of a not indicated amount of biotinylated TNF followed by detection of cell-bound TNF using streptavidin-FITC. Indicated concentrations are based on the assumption of a volume of 200 µl which is typical for this type of assay*.

*^e^In a volume not indicated in the manuscript, cells were preincubated with 5,000 ng PGRN followed by addition of 50 ng biotinylated TNF. Cell-bound TNF was detected using streptavidin-FITC. Indicated concentration is based on the assumption of a volume of 200 µl which is typical for this type of assay*.

## Results

**Figure 1:**
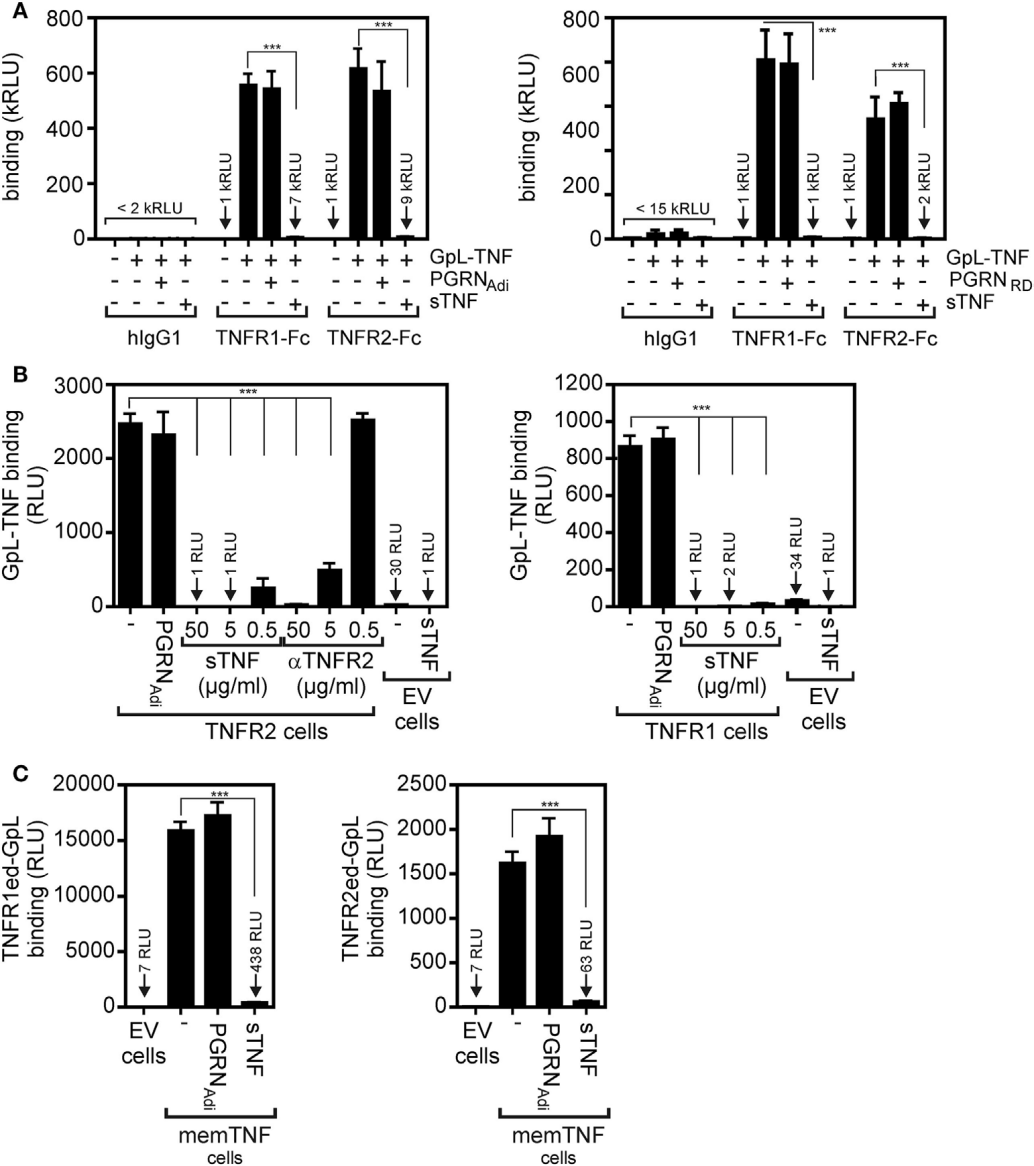
Preincubation with purified progranulin (PGRN) samples does not interfere with tumor necrosis factor (TNF)-tumor necrosis factor receptor-1 (TNFR1) and TNF–TNFR2 interaction in cell-free and cellular binding studies. **(A)** TNFR1-Fc and TNFR2-Fc or an irrelevant human IgG1 (Rituximab) were immobilized to black enzyme-linked immunosorbent assay (ELISA) plates. Where indicated wells were preincubated with 25 µg/ml untagged PGRN from Adipogen (PGRN_Adi_) or 25 µg/ml myc-6xHis-tagged PGRN from R&D Systems (PGRN_RD_) for 1 h. GpL-TNF was then added to reach a concentration of 10 ng/ml and finally bound GpL-TNF was quantified by measuring its GpL activity. As positive control for successful competitive binding inhibition groups were included, where 10 µg/ml of soluble TNF (sTNF) have been added instead of PGRN. **(B)** Human embryonal kidney cells 293 (HEK293) cells were transfected with empty vector (EV) or expression plasmids encoding a deletion mutant of TNFR1, where the death domain has been replaced by yellow fluorescence protein (YFP) (TNFR1) or deletion mutant of TNFR2, where the TRAF2 binding site has been replaced again by YFP (TNFR2). Next day, aliquots of cells (1 × 10^6^) were preincubated with 500, 5,000, or 50,000 ng/ml of sTNF or 50,000 ng/ml PGRN_Adi_ for 2 h at 37°C or remained untreated. Binding studies were performed in technical triplicates with 20 ng/ml GpL-TNF. In the experiment with TNFR2-transfected cells, a group was pretreated with 20 µg/ml of a blocking TNFR2-specific antibody (αTNFR2). Please note, GpL-TNF binding of EV-transfected cells in the presence and absence of an excess of sTNF defines the low endogenous expression of TNF receptors which was about 1–3% of the ectopically expressed receptors. **(C)** EV-transfected control cells and membrane TNF (memTNF) expressing transfectants were incubated with 100 ng/ml of TNFR1ed-GpL or TNFR2ed-GpL and mixtures of these GpL variants with 2,000 ng/ml sTNF or 2,000 ng/ml PGRN_Adi_. After 90 min, unbound molecules were removed and specific binding was again obtained by subtracting non-specific binding (EV transfectants) from total binding (memTNF transfectants). Please be aware, the fact that specific binding of TNFR1ed-GpL is app. Tenfold higher than those of TNFR2ed-GpL reflects the fact that soluble monomeric TNFR1 has much higher affinity for TNF than soluble TNFR2 molecules and that non-saturating soluble receptor concentrations have been used in this competition assays. ***p < 0.0001.

In SPR experiments, in which PGRN binding to the soluble monomeric extracellular domains of TNFR1 and TNFR2 monomers adsorbed to a sensor chip has been investigated, Tang et al. identified PGRN as a high affinity ligand of TNFR1 and TNFR2 with K_D_-values of 1.77 and 1.52 nM (7). These K_D_-values were close or even much lower than those of sTNF (7.94 nM for TNFR1; 910 nM for TNFR2) measured in the same study with the same methodology (7). It is, however, important to note in this context that on intact cells the affinities of sTNF for its two receptors are much higher and are in the range of 0.02–0.65 nM for TNFR1 and 0.08–0.4 nM for TNFR2 [e.g., Ref. (25–32)]. Since PGRN was furthermore reported to act as a competitive inhibitor of sTNF binding, we evaluated the ability of recombinant purified PGRN to inhibit binding of a *Gaussia princeps* luciferase (GpL) fusion protein of soluble TNF (GpL-TNF) to TNFR1 and TNFR2. The luciferase from the mesopelagic copepod *Gp* is a secreted, small (19 kDa), monomeric luciferase with superior brightness. GpL fusion proteins offer, therefore, an exquisite sensitivity and a for several orders of magnitude linear signal strength (33). In particular, we have demonstrated that fusion of a GpL domain to sTNF neither affects sTNF activity nor sTNF receptor binding (32, 34). TNF-GpL is, therefore, ideally suited to evaluate competitive inhibitors of TNF–TNFR1 and TNF–TNFR2 interaction in cell-free and cellular binding studies. In a prototypical cell-free competition assay with plastic-bound Fc fusion proteins of the TNFR1 and TNFR2 ectodomains (TNFR1ed-Fc and TNFR2ed-Fc), we obtained no evidence for a significant inhibition of GpL-TNF binding to the two TNF receptors by a >1,000 fold excess of commercially available PGRN samples (Figure 1A). Since it has been argued that PGRN of some suppliers does not interact with TNFR1 and TNFR2, we used PGRNs from Adipogen (PGRN_Adi_) and R&D Systems (PGRN_RD_) which have been cited in the studies reporting direct TNFR1/2–PGRN interaction (Table 1). Next, we analyzed the effect of PGRN on GpL-TNF binding to cells transfected with expression plasmids encoding TNFR1 and TNFR2. To prevent disturbance by signaling related effects of the overexpressed receptors (in the case of TNFR1 there is, for example, apoptosis induction after transient expression!), we used a cytosolic deletion mutant of TNFR1 in which the death domain of the molecule has been replaced by the yellow fluorescence protein (YFP) and a TNFR2 variant in which the intracellular binding site for the signaling molecule TNF receptor associated factor-2 (TRAF2) has been substituted by YFP, too. Specific binding of 20 ng/ml (=200 pM) GpL-TNF to the transiently expressed TNFR1 and TNFR2 molecules was >800- and >2,400-fold over the unspecific background of mock-transfected control cells pretreated with a 50,000 ng/ml of unlabeled sTNF. Preincubation of the TNF receptor transfectants with 50,000, 5,000, and 500 ng/ml of sTNF diminished specific binding of GpL-TNF to both receptors completely, for >99 or >90% (Figure 1B). Preincubation of the TNFR1/2 transfectants with 50,000 ng/ml PGRN from Adipogen, however, showed no significant inhibition of GpL-TNF binding (Figure 1B). We obtained similar negative results with two other batches of PGRN from the same supplier and with PGRN from R&D systems (Figure S1 in Supplementary Material). Reciprocal binding studies with membrane TNF expressing cells and GpL fusion proteins of soluble TNFR1 and TNFR2 variants containing the ectodomains (ed) of these receptors (TNFR1ed-GpL and TNFR2ed-GpL) yielded comparable results. Specific binding of TNFR1ed-GpL and TNFR2ed-GpL to membrane TNF expressing HEK293 cells was >2,000- and >200-fold over background (Figure 1C). Pretreatment of the soluble GpL-receptor molecules with an excess of sTNF again reduced specific binding for more than 95% while preincubation with PGRN_Adi_ showed again no significant inhibitory effect on TNF–TNFR1/2 interaction (Figure 1C).

**Figure 2:**
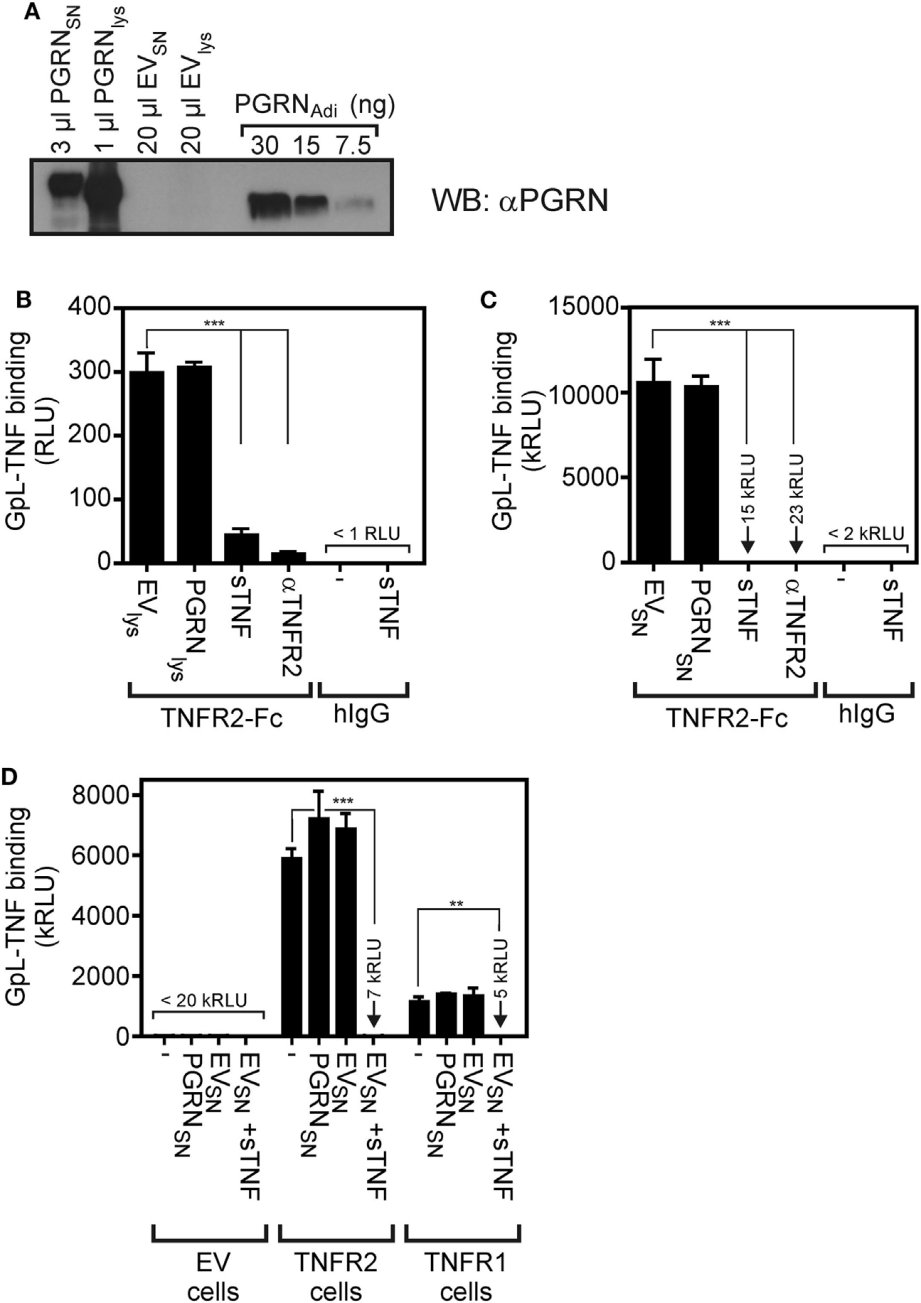
Preincubation with untagged human embryonal kidney cells 293 (HEK293) cell-derived progranulin (PGRN) does not interfere with tumor necrosis factor (TNF)-tumor necrosis factor receptor-1 (TNFR1) and TNF–TNFR2 interaction in cell-free and cellular binding studies. **(A)** HEK293 cells were transfected with empty vector (EV) or an expression plasmid encoding non-tagged PGRN. The indicated volume of supernatants (SNs) and cell lysates derived from these transfectants along with recombinant PGRN_Adi_ (30, 15, and 7.5 ng) as standard were analyzed by Western blotting for the presence of PGRN and estimation of PGRN concentration. PGRN concentrations reached approximately 10,000 ng/ml in the SN of PGRN transfected cells (PGRN_SN_) and approximately 30,000 ng/ml in the corresponding cell lysate (PGRN_lys_). There was no detectable endogenous PGRN expression neither in the SN (EV_SN_) nor the lysate (EV_lys_) of EV-transfected cells. **(B,C)** TNFR2-Fc or, as a control for unspecific binding, IgG1 was immobilized to black enzyme-linked immunosorbent assay plates. Lysates **(B)** and SNs **(C)** of PGRN and EV-transfected cells [PGRN_lys_ and EV_lys_ **(B)**, PGRN_SN_ and EV_SN_ **(C)**] were added for 1 h before the specific binding of 50 ng/ml and GpL-TNF was determined in triplicates. Where indicated immobilized TNFR2-Fc was pretreated for 1 h with 2,000 ng/ml sTNF or 20 µg/ml of a neutralizing TNFR2-specific antibody (αTNFR2). **(D)** HEK293 transfectants expressing TNFR1 or TNFR2 along with control HEK293 cells transfected with EV were preincubated for 1 h with pure PGRN_SN_, pure EV_SN_, and pure EV_SN_ with and without supplementation with 10,000 ng/ml sTNF. After preincubation, cells were incubated in triplicates with 10 ng/ml GpL-TNF at 37°C for 1 h and finally cell-bound GpL activity was determined. ***p < 0.0001; **p < 0.001.

To minimize possible unknown negative effects of the purification process and storage conditions of the commercially obtained PGRN samples on their ability to bind to TNFR1 and TNFR2, we used next cultures supernatants (SNs) and lysates of HEK293 cells transiently transfected with an expression plasmid encoding human PGRN. The human cell line HEK293 has been used here because HEK293 cells not only ensure high transfection efficiency, but has also used for PGRN production by groups reporting PGRN–TNFR interaction (7, 10, 23). Western blotting with a PGRN-specific antibody and PGRN_Adi_ as mass standard showed that PGRN production in the SN (PGRN_SN_) reached up to approximately 10,000 ng/ml and lysates of PGRN expressing cells (PGRN_lys_) contained approximately 30,000 ng/ml of the protein (Figure 2A). Noteworthy, PGRN_lys_ somewhat faster in the gel than PGRN_SN_ and both PGRN_lys_ and PGRN_SN_ migrated slower (approximately 85–90 kDa) compared to the PGRN_Adi_ standard (70–80 kDa, Adipogen data sheet indicates 74 kDa) which was derived of HEK293 cells, too. Thus, PGRN of different sources appears to be differentially modified (e.g., by glycosylation). The sizes of PGRN_lys_ and PGRN_SN_ are in accordance with the literature typically indicating a size of 88 kDa for PGRN. Next, we subjected PGRN_lys_ and PGRN_SN_ along with corresponding samples of empty vector (EV)-transfected cells to competitive binding studies with plate-bound TNFR2-Fc and GpL-TNF. There was again no evidence for an interference of PGRN with the interaction of GpL-TNF and TNFR2. Neither pretreatment with PGRN_lys_ nor with PGRN_SN_ showed a significant inhibitory effect on GpL-TNF binding to TNFR2-Fc (Figures 2B,C). In contrast, lysates and SNs of EV-transfected cells (EV_lys_ and EV_SN_) supplemented with 2,000 ng/ml soluble TNF showed efficient inhibition of binding of GpL-TNF (Figures 2B,C). Similar results were obtained with plastic-bound TNFR1-Fc instead of TNFR2-Fc (Figure S2 in Supplementary Material). There was also no significant inhibitory effect of PGRN_SN_ on TNF–TNFR2 interaction on intact cells (Figure 2D; Figure S3 in Supplementary Material). Please note, cellular binding studies with the PGRN containing cell lysates (PGRN_lys_) were not possible due to the cell lytic effects of the lysis buffer. It should also be stressed that the lysis buffer used was prepared according to Tang et al. reporting PGRN-TNFR2 co-immunoprecipitation in this buffer (7). To evaluate the effect of PGRN on TNF–TNFR1/2 interaction in a second independent cellular model, we performed competitive binding studies with HeLa-TNFR2 cells. HeLa-TNFR2 is a stable HeLa transfectant expressing in addition to endogenous TNFR1 also TNFR2 due to stable transfection (35). Despite their obvious different degree of modification (Figure 2A), the various PGRN variants (PGRN_Adi_, PGRN_RD_, and PGRN_SN_) had no effect on GpL-binding. In contrast, pretreatment with sTNF or Flag-LTα (F-LTα) inhibited GpL-binding for >99 and >98% indicating efficient blockade of TNFR1 and TNFR2 (Figure S4 in Supplementary Material).

**Figure 3:**
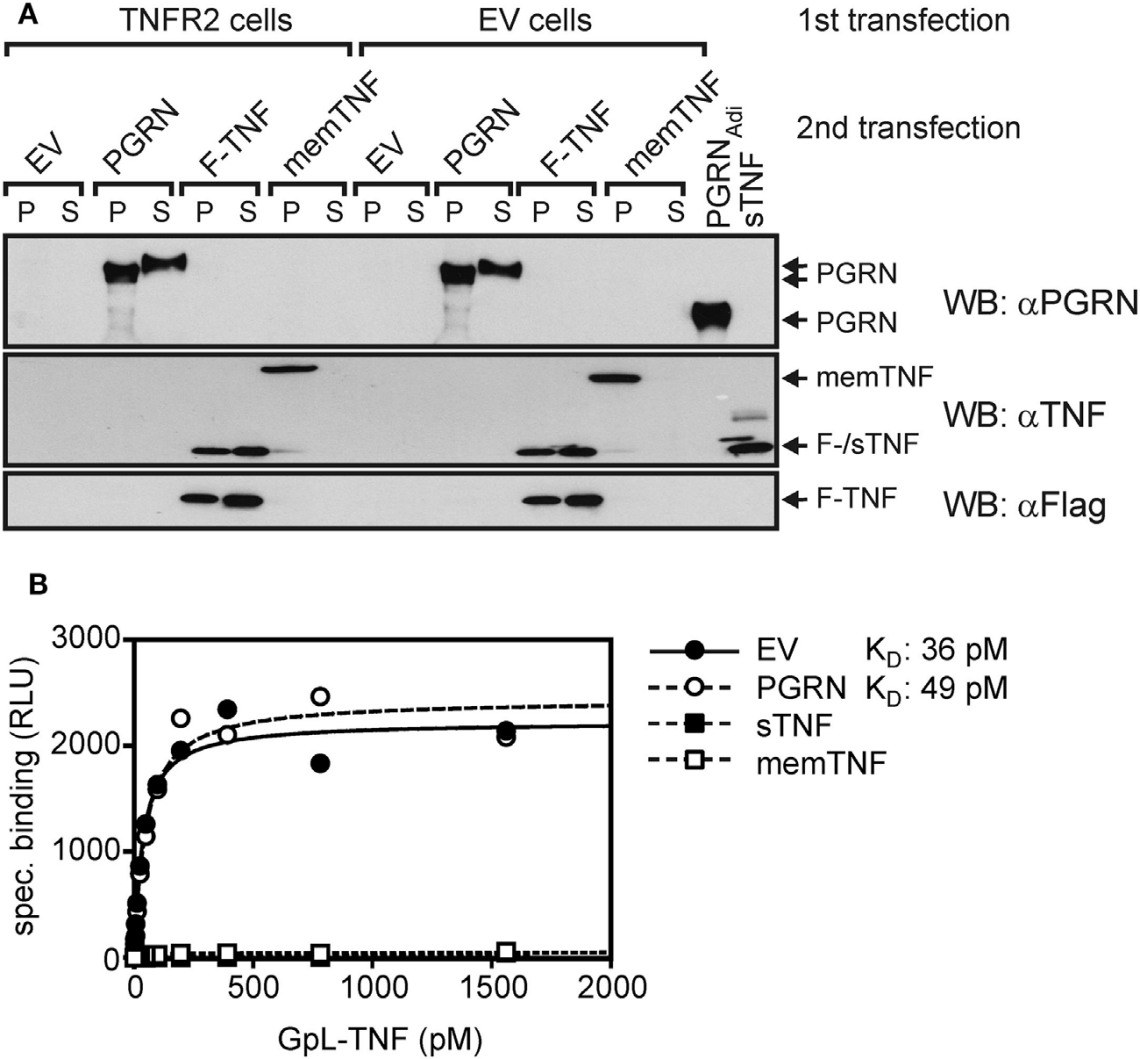
*Gaussia princeps* luciferase (GpL)-tumor necrosis factor (TNF) binding to cells with endogenous coexpression of tumor necrosis factor receptor-2 (TNFR2) and progranulin (PGRN). **(A)** Human embryonal kidney cells 293 (HEK293) cells were transfected (first transfection) with empty vector (EV) or an expression vector encoding TNFR2, where the TNF receptor associated factor 2 (TRAF2) binding site has been replaced by yellow fluorescence protein (YFP) (TNFR2). The following day, transfecfed cells were split into four aliquots which were transfected a second time (second transfection) with expression plasmids encoding PGRN, membrane TNF (memTNF), soluble Flag-tagged TNF (F-TNF), or EV. After an additional day, aliquots of 30,000 cells (P) and 15 µl SN (S) were analyzed by Western blotting with anti-PGRN, anti-TNF, and anti-Flag along with 100 ng PGRN_Adi_ and 100 ng purified untagged soluble TNF (sTNF). **(B)** Equilibrium binding studies were performed with the indicated concentrations of GpL-TNF. Specific binding of GpL-TNF in the presence of PGRN (second transfection PGRN), membrane TNF (second transfection memTNF), and Flag-TNF (second transfection F-TNF) or the absence of an potential modulator (second transfection EV) was obtained by subtracting unspecific binding values (first transfection EV) from the corresponding total binding values (first transfection TNFR2). Specific binding values were fitted by non-linear regression analysis to a single binding site type of interaction by help of the GraphPad Prism 5 software.

To further minimize possible influencing variables affecting PGRN–TNF receptor interaction, we secondarily transfected TNFR2 transfectants with expression plasmids encoding PGRN (PGRN), soluble TNF (sTNF), or membrane TNF (memTNF), and analyzed the transfected cells finally again for GpL-TNF binding. As expected transfection of plasmids encoding sTNF or memTNF resulted in strong reduction of GpL-TNF binding to TNFR2 (Figures 3A,B). Once again PGRN expression failed to have an effect on TNF–TNFR2 interaction despite robust expression yielding approximately 3,000 ng/ml PGRN in the cell culture SN and cell-associated expression comparable to those of memTNF (Figure 3A). Coexpression of PGRN neither showed an effect on the number of binding sites for GpL-TNF nor on the K_D_-value of the interaction of GpL-TNF and TNFR2 (Figure 3B). In this simplified and highly sensitive experimental setting, both PGRN and its potential binding partner TNFR2 were directly expressed by the cells in the assay. This maximally rules out that experimental handling of the two possible binding partners or their purification can affect or change their interaction. In comparable experiments where TNFR1 has been transiently expressed along with the sTNF and PGRN encoding expression plasmids, similar results were obtained (Figure S5 in Supplementary Material).

**Figure 4:**
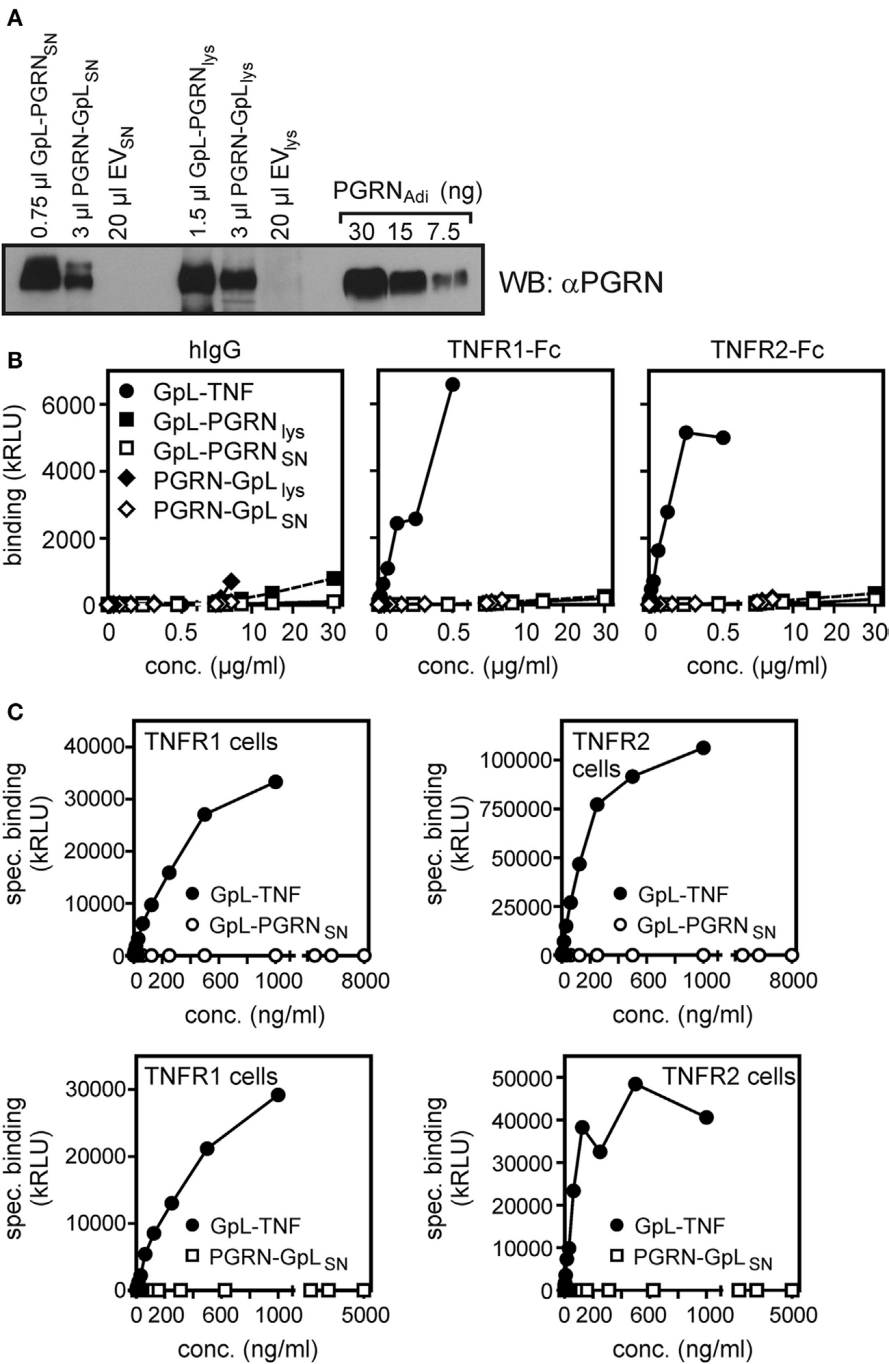
*Gaussia princeps* luciferase (GpL) fusion proteins of progranulin (PGRN) show no relevant binding to tumor necrosis factor receptor-1 (TNFR1) or TNFR2. **(A)** Human embryonal kidney cells 293 (HEK293) cells were transiently transfected with expression plasmids encoding GpL-PGRN (GpL-PGRN), PGRN-GpL (PGRN-GpL), or empty vector (EV). GpL-PGRN concentrations in supernatants (SNs) and cell lysates were determined by help of a GpL fusion protein of known concentration. SNs and cell lysates, containing approximately 100 ng PGRN-GpL or GpL-PGRN along with 100 ng PGRN_Adi_, were subjected to Western blotting with a PGRN-specific antibody to verify the integrity of the PGRN GpL fusion proteins. **(B)** TNFR1-Fc, TNFR2-Fc or, as a control for unspecific binding, hIgG1 were immobilized to black enzyme-linked immunosorbent assay plates. Lysates and SN of the GpL-PGRN (GpL-PGRN_lys_ and GpL-PGRN_SN_) and PGRN-GpL (PGRN-GpL_lys_ and PGRN-GpL_SN_) transfected cells and GpL-tumor necrosis factor (TNF) were added for 1 h and binding was determined in triplicates. **(C)** TNFR1 and TNFR2 expressing transfectants (total binding) and EV-transfected HEK293 cells (non-specific binding) were subjected to equilibrium binding studies with the indicated GpL fusion proteins. Specific binding (= total − non-specific binding) values were fitted by non-linear regression analysis to a single binding site type of interaction by help of the GraphPad Prism 5 software.

Finally, we generated PGRN fusion proteins with an N- and a C-terminal GpL domain (GpL-PGRN and PGRN-GpL) and investigated their binding to TNFR1 and TNFR2. While 4–250 ng/ml GpL-TNF showed significant binding to plastic-bound TNFR1-Fc and TNFR2-Fc, there was no significant binding with lysates and SNs of GpL-PGRN (GpL-PGRN_lys_ and GpL-PGRN_SN_) and PGRN-GpL (PGRN-GPL_lys_ and PGRN-GpL_SN_) expressing HEK293 cells despite using concentration of up to 5,000–30,000 ng/ml (Figures 4A,B). Likewise, there was no relevant specific binding of GpL-PGRN_SN_ and PGRN-GpL_SN_ to TNFR1 and TNFR2 transfected cells (Figure 4C). Although, one cannot fully rule out that an authentic N- or C-terminus of PGRN is important for its putative interaction with TNF receptors, this appears unlikely because PGRN activity has been reported with various N- and C-terminally tagged variants (Table 1).

## Discussion

A variety of studies demonstrated that PGRN can inhibit TNF-induced cellular activities. The identification of PGRN as a protein that “*directly binds to TNFR*” and causes “*dose-dependent inhibition of TNF*α *binding to TNFR1 and TNFR2*” (7) offered a simple and straightforward explanation of the inhibitory effects of PGRN on TNF activity at the molecular level. However, two independent groups failed to reproduce PGRN binding to TNFR1 (20, 21). Two other groups found furthermore no evidence for an inhibitory action of PGRN on TNF-induced signaling or even reported enhanced TNF activity (9, 22). It has been suggested that this was due to “problematic” PGRN preparations and technical differences in the cell-free analysis of PGRN–TNFR1/2 interaction by SPR (24). Indeed, the PGRN variants used by the various groups differed with respect to the position and nature of tags or were from different suppliers (Table 1). To avoid the possible impact of the commercial source, purification procedures or tagging and to maximally reduce the relevance of “technical” factors, we analyzed the inhibitory effect of PGRN on receptor binding of TNF in cellular binding studies at 37°C in normal culture medium not only with PGRN from commercial sources, but also with fresh, non-purified untagged, and thus fully authentic PGRN released from transiently transfected HEK293 cells. Neither, PGRN samples from Adipogen and R&D Systems, which has been used in reports demonstrating PGRN–TNF receptor interaction, nor HEK293-derived SNs containing untagged PGRN showed an inhibitory effect on binding of a GpL-TNF fusion protein to plastic-bound or cell-expressed TNFR1/2 (Figures 1 and 2; Figures S1, S2, and S4 in Supplementary Material). In the experiments with commercially available purified PGRN samples, we used concentrations up to 25 and 50 µg/ml (approximately 280 and 560 nM) and the HEK293-derived PGRN containing SNs reached concentrations of around 30 µg/ml, too (Figure 2A). This was not only a huge excess over GpL-TNF (MW 100,000), which was applied with 2–50 ng/ml (approximately 0.02–0.5 nM), but also far higher than the PGRN concentrations used in the literature to modulate TNF signaling, or than the PGRN levels in the synovial fluid of patients suffering on rheumatoid arthritis (68 ng/ml) or malignant lymphomas (91.3 ng/ml) (36, 37). Intriguingly, expression of PGRN in TNFR2 expressing (Figure 3) or in TNFR1 expressing cells (Figure S5 in Supplementary Material) showed no effect on TNF binding. In an independent approach, we looked also for direct binding of PGRN to plastic-bound and cell-expressed TNFR1 and TNFR2. For this purpose, we used non-purified HEK293-derived variants of PGRN with an N- or C-terminal GpL-flag reporter domain. With none of these two variants we found evidence for significant TNFR1 or TNFR2 binding (Figure 4). Since various PGRN variants (Table 1) successfully used in the literature to study the PGRN-TNF crosstalk also carried N- and/or C-terminal tags including a His-tag which has the potential to interfere with the numerous Cys residues in PGRN, it appears unlikely that these negative data have been caused by the sole use of a tag.

Our studies are mainly based on the use of a GpL-fusion protein of TNF. One possibility for the failure of PGRN to block GpL-TNF binding to TNFR1 and TNFR2, was that the GpL domain might, instead, directly interact with PGRN, and artifactually prevent it from interacting with the TNF receptors. This can, however, be ruled out because we used up to >1,000-fold molar excess of PGRN in our PGRN/GpL-TNF competition experiments. Therefore, even if the GpL domain of GpL-TNF did bind irreversibly to PGRN, a huge surplus of “GpL-free” PGRN would have been available in our experiments to block TNFR1/TNFR2 binding by GpL-TNF (or by GpL-TNF in complex with PGRN).

In sum, we found no evidence for a direct and TNF binding-competing interaction of PGRN and TNF receptors even not in experimental settings, where PGRN and TNF receptors were expressed directly by the cells in the assay and where their potential interaction can thus not be affected by unknown factors related to experimental processing (Figure 3; Figure S5 in Supplementary Material). Our results convincingly argue against a direct generally occurring prototypic PGRN–TNFR1/2 interaction, but of course cannot rule out complex interaction scenarios, requiring for yet unknown additional factors or chemical or biological modification of PGRN. We want to stress in this context that already the studies reporting direct PGRN–TNFR1/2 interaction give indications which challenge the idea that PGRN acts as a prototypic ligand binding-blocking interaction partner of TNFR1 and TNFR2.

First, in the initial study describing PGRN as a high-affinity ligand for TNFR1 and TNFR2, Tang et al. reported affinities of 1.77 and 1.52 nM for these receptors (7). However, despite the strong affinities of the PGRN–TNFR1 and PGRN–TNFR2 interactions, excessive high concentrations of PGRN (75,000 ng/ml = 833 nM) were required to see inhibitory effects on binding of sTNF to TNFR1 and TNFR2 and this although TNF receptor activities were inhibited at much lower PGRN concentrations (Table 2). Likewise, in various follow up studies the fluorescence-activated cell sorting (FACS)- and enzyme-linked immunosorbent assays (ELISA)-based demonstration of PGRN binding to TNFR1/2 in competition experiments with TNF required again concentrations in the μM instead of nM range while modulation of TNF signaling was evident at >two orders of magnitude lower PGRN concentrations (Table 2). Of course, the huge discrepancy in the reported PGRN concentrations required to inhibit TNF signaling and to block TNF binding is not compatible with the mode of action of a simple competitive inhibitor. Second, Tian et al. analyzed TNFR2-expressing Raw264.7 and THP-1 cells for TNF binding (50 ng/ml) by FACS and reported that the ability of a high excess of PGRN (5,000 ng) to reduce sTNF binding was diminished at higher cell densities (10). Such a cell density/receptor number dependency of the ability of PGRN to interfere with TNF binding is again not straightforwardly compatible with competitive binding inhibition. Third, PGRN enhances TNF-induced TNFR2-mediated proliferation and suppressive activity of regulatory T cells (9) and PGRN-induced Akt signaling has found to be inhibited by neutralizing TNFR2 antibodies (15, 18). Both observations again argue against competitive inhibition of TNF binding by PGRN.

## Conclusion

Two independent studies failed to demonstrate inhibition of TNF binding to TNF receptors by PGRN (20, 21). Our results obtained in highly sensitive cellular binding studies with two commercially available PGRN samples and GpL-tagged and untagged PGRN containing cell culture SNs also gave no evidence for high affinity and/or competitive PGRN binding to TNFR1 and TNFR2. Thus, it is obvious that the putative direct and competitive interactions of PGRN with the two TNF receptors are not robust and straightforwardly reproducible. Future studies must identify the factors or modifications which enable PGRN to bind TNF receptors. Till then we recommend to be careful in assigning inhibitory effects of PGRN on TNF function to competitive inhibition of TNF–TNFR1/2 interaction without direct concomitant experimental evaluation.

## Materials and Methods

### Reagents and Cell Lines

Progranulin was purchased from Adipogen, Liestal, Switzerland (untagged protein, #AG-40A-0188Y) and R&D Systems, Wiesbaden, Germany (C-terminally myc-6xHis-tagged, #). The expression vector (pCMV6-XL5) encoding untagged human PGRN (Ac. No.: NM_002087) was from Origene, Rockville, MD, USA (#SC118822). The anti-PGRN mouse monoclonal antibody C-11 and the TNF-specific goat IgG N-19 were from Santa Cruz Biotechnology, Dallas, USA (C-11: #sc-377036; N-19 #sc-1350). The pEF-BOS-based expression vector encoding membrane TNF have been described elsewhere (31). TNFR1-Fc was from R&E Systems, Wiesbaden, Germany; TNFR2-Fc (Enbrel) was from Pfizer, the TNF-specific antibody Humira was from AbbVie (Wiesbaden, Germany), and soluble TNF was a kind gift of Prof. Daniela Männel (University of Regensburg). The TNFR1-specific antibody H398 was kindly provided by Prof. Klaus Pfizenmaier (University of Stuttgart). Production, characterization, and use of GpL-Flag-TNC-TNF (abbreviated in the study as GpL-TNF) and Flag-LTα have been described in detail elsewhere (32, 34). The Flag tag in GpL-Flag-TNC-TNF was introduced for affinity purification and the tenascin-C trimerization domain stabilizes the trimeric nature of the TNF molecule. HEK293 cells (ATCC) were cultivated in RPMI1640 medium (Sigma-Aldrich, Schnelldorf, Germany) supplemented with 10% fetal calf serum (Gibco—Thermo Fisher Scientific, Darmstadt, Germany). The Flag tag-specific antibody was again from Sigma-Aldrich (Schnelldorf, Germany). HeLa cells stably transfected with TNFR2 (HeLa-TNFR2) have been described elsewhere (35). Typical FACS results of TNFR2 and TNFR1 expression of HeLa-TNFR2, HEK293, and HEK293 cells transiently expressing TNFR1/2 variants are shown in Figure S6 in Supplementary Material.

### Molecular Cloning and Expression of Recombinant Proteins

The expression vector encoding soluble Flag-TNF was generated by replacement of the TRAILR2 encoding part in PS435 (kind gift of Prof. Pascal Schneider, University of Lausanne), a pCR3.1-based expression vector (Invitrogen—Thermo Fisher Scientific, Darmstadt, Germany) encoding the human Ig leader followed by a Flag-tag and TRAILR2, with a DNA amplicon encoding aa 85–223 of human TNF (ac. no.: NP000585). The TNFR1ed-GpL and TNFR2ed-GpL encoding expression plasmids are also based on pCR3.1 and encode expression cassettes comprising the ectodomain of TNFR1 (aa 1–211 of ac. no.: M58286.1) or TNFR2 (aa 1–257 of ac. no.: M55994.1) followed by the Flag epitope and aa 18–185 of ac. no.: GM037681 encoding mature, thus leader free GpL. Two aa insertions (GSAGEF and LE) resulting from molecular cloning furthermore separate the Flag tag from the receptor and GpL parts, respectively. The GpL-PGRN encoding expression plasmid is also a pCR3.1 derivative and encodes GpL including its leader sequence followed by a Flag tag and aa 21–593 of human PGRN whereby the Flag epitope is connected with the GpL and the PGRN domain by a five aa (SGAGS) and a two aa (EF) insertion.

Recombinant proteins were produced in HEK293 cells by transient transfection of the expression plasmids described above. For this purpose, the medium of tissue culture dishes with confluent HEK293 cells was replaced by 15 ml of serum-free RPMI 1640 medium containing penicillin–streptomycin. For each culture, 2 ml of RPMI 1640 medium containing 12 µg of the expression plasmid of interest were prepared and supplemented dropwise and under vortexing with 36 µl of a 1 mg/ml polyethylenimine (PEI, Polysciences Europe, Hirschberg, Germany). After 15 min at room temperature, the plasmid/PEI solution was added and transferred to the HEK293 cells. Next day, the serum-free plasmid/PEI-containing medium was replaced by fresh RPMI 1640 medium containing 2% FCS and penicillin–streptomycin. After 4–6 days, SNs were collected and cellular debris was removed by centrifugation (10 min, 4,630 *g*). The resulting PGRN containing SN (PGRN_SN_) was directly used for experiments or after dilution in cell culture medium. To obtain cell-associated PGRN (PGRN_lys_), correspondingly transfected cells were harvested 48 or 72 h post transfection and lysed in RIPA buffer (Cell Signaling, Leiden, Netherlands). Finally, concentration of the protein of interest was evaluated by Western blotting and an appropriate protein mass standard and/or by measuring the activity of the GpL domain. The complete Western blots of the cuttings shown in Figures 2A, 3A and 4A are documented in Figure S7 in Supplementary Material.

### Binding Studies

For cell-free binding studies with plastic surface-immobilized protein, solutions (2 µg/ml in PBS or 0.1 M carbonate buffer) of the purified protein of interest [TNFR1-Fc, TNFR2-Fc, PGRN, Rituximab (Roche, Basel, Switzerland), a CD20-specific human IgG1 molecule, as a negative control for TNFR1/2-Fc] were subjected to black high bind ELISA plates (Greiner, Frickenhausen, Germany). After overnight incubation at 4°C and three washes with PBS Tween, remaining free binding sites were saturated by incubation (1 h, room temperature) with blocking buffer (10% FCS in PBS). After three washing cycles with PBS Tween, the actual binding studies were performed. In the case of equilibrium binding studies wells were incubated for 2 h with increasing concentrations of the GpL fusion protein of interest at room temperature. Unbound protein was then removed by five wash cycles with PBS Tween and finally well-associated luciferase activity was determined (see below). Values for non-specific binding were derived from wells coated with control protein or with coating buffer only and were subtracted from the corresponding total binding values obtained from the wells coated with the protein of interest to obtain specific binding values. In the case of competition binding studies, wells were treated for 0.5–1 h with increasing concentrations of the potential inhibitor (PGRN variants, sTNF, Flag-LTα) or remained untreated before the GpL fusion protein was added for an additional hour. Finally, wells were again washed five times and used for quantification of bound luciferase activity.

For cellular binding studies, HEK293 cells were transiently transfected with expression plasmids encoding the protein of interest and EV. Next day, cells were divided into the required number of aliquots of 0.5–1 × 10^6^ cells in 150 µl RPMI 1640 medium with 10% FCS. Where indicated, cells were then pretreated at 37°C for 1 h with potential antagonistic proteins (sTNF, Flag-LTα, and PGRN variants), otherwise cells remained untreated. Cells were then supplemented with the GpL fusion protein of interest and after an additional incubation period of 1 h, unbound proteins were removed (five washes with PBS). Finally, cells were collected in 50 µl of RPMI 1640 medium with 0.5% FCS to quantify the remaining cell-bound GpL fusion protein molecules. Binding values derived of EV-transfected cells were considered as non-specific binding and binding values obtained from the transfectants expressing the protein of interest were considered as total binding. Please note, the expression levels observed after transfection of expression plasmids encoding TNFR1 and TNFR2 were regularly >100-fold higher than those of endogenously expressed TNFR1. There was no evidence for endogenous expression of TNF in the HEK293 cells.

*Gaussia princeps* luciferase activity was measured with the *Gaussia* luciferase Assay Kit (New England Biolabs, Frankfurt, Germany) essentially as described by the supplier. After starting the reaction by adding substrate-buffer solution, light emission was immediately (<10 s) quantified (Lucy 2 or a LUmo Luminometer; both Anthos Labtec Instruments) to minimize errors due to the decay of GpL activity. Please note, the LUmo Luminometer has a much higher sensitivity compared to the Lucy 2 luminometer. Data are reported as mean ± SEM and were analyzed by Bonferroni’s test or were analyzed with the “nonlinear regression to a one-site specific binding curve” or the “nonlinear regression to a one-site competitive binding curve” function of the GraphPad Prism5 software.

## Author Contributions

HW designed the study and prepared the manuscript. IL and SF performed the experiments and contributed to study design. All authors read and approved the final manuscript.

## Conflict of Interest Statement

The authors declare that the research was conducted in the absence of any commercial or financial relationships that could be construed as a potential conflict of interest.

## Abbreviations

ELISA, enzyme-linked immunosorbent assays; FACS, fluorescence-activated cell sorting; GpL, *Gaussia princeps* luciferase; HEK293, human embryonal kidney cells 293; KD, equilibrium dissociation constant; PGRN, progranulin; SPR, surface plasmon resonance; TNF, tumor necrosis factor; TNFR1/2, tumor necrosis factor receptor-1/2.

## References

[1] Wajant H Pfizenmaier K Scheurich P. Tumor necrosis factor signaling. Cell Death Differ (2003) 10:45–65. 10.1038/sj.cdd.4401189 12655295

[2] Locksley RM Killeen N Lenardo MJ The TNF and TNF receptor superfamilies: integrating mammalian biology. Cell (2001) 104:487–501. 10.1016/S0092-8674(01)00237-9 11239407

[3] Banner DW D’Arcy A Janes W Gentz R Schoenfeld HJ Broger C Crystal structure of the soluble human 55 kd TNF receptor-human TNF beta complex: implications for TNF receptor activation. Cell (1993) 73:431–45. 10.1016/0092-8674(93)90132-A 8387891

[4] Mukai Y Nakamura T Yoshikawa M Yoshioka Y Tsunoda S Nakagawa S Solution of the structure of the TNF-TNFR2 complex. Sci Signal (2010) 3:ra83. 10.1126/scisignal.2000954 21081755

[5] Cenik B Sephton CF Kutluk Cenik B Herz J Yu G. Progranulin: a proteolytically processed protein at the crossroads of inflammation and neurodegeneration. J Biol Chem (2012) 287:32298–306. 10.1074/jbc.R112.399170 22859297 PMC3463300

[6] Kao AW McKay A Singh PP Brunet A Huang EJ. Progranulin, lysosomal regulation and neurodegenerative disease. Nat Rev Neurosci (2017) 18:325–33. 10.1038/nrn.2017.36 28435163 PMC6040832

[7] Tang W Lu Y Tian QY Zhang Y Guo FJ Liu GY The growth factor progranulin binds to TNF receptors and is therapeutic against inflammatory arthritis in mice. Science (2011) 332:478–84. 10.1126/science.1199214 21393509 PMC3104397

[8] Zhao YP Tian QY Frenkel S Liu CJ. The promotion of bone healing by progranulin, a downstream molecule of BMP-2, through interacting with TNF/TNFR signaling. Biomaterials (2013) 34:6412–21. 10.1016/j.biomaterials.2013.05.030 23746860 PMC3713419

[9] Hu Y Xiao H Shi T Oppenheim JJ Chen X Progranulin promotes tumour necrosis factor-induced proliferation of suppressive mouse CD4(+) Foxp3(+) regulatory T cells. Immunology (2014) 142:193–201. 10.1111/imm.12241 24383743 PMC4008227

[10] Tian Q Zhao Y Mundra JJ Gonzalez-Gugel E Jian J Uddin SM Three TNFR-binding domains of PGRN act independently in inhibition of TNF-alpha binding and activity. Front Biosci (Landmark Ed) (2014) 19:1176–85. 10.2741/4274 24896343 PMC4410860

[11] Mundra JJ Jian J Bhagat P Liu CJ. Progranulin inhibits expression and release of chemokines CXCL9 and CXCL10 in a TNFR1 dependent manner. Sci Rep (2016) 6:21115. 10.1038/srep21115 26892362 PMC4759551

[12] Zhao YP Liu B Tian QY Wei JL Richbourgh B Liu CJ Progranulin protects against osteoarthritis through interacting with TNF-alpha and beta-catenin signalling. Ann Rheum Dis (2015) 74:2244–53. 10.1136/annrheumdis-2014-205779 25169730 PMC4408266

[13] Kawase R Ohama T Matsuyama A Matsuwaki T Okada T Yamashita T Deletion of progranulin exacerbates atherosclerosis in ApoE knockout mice. Cardiovasc Res (2013) 100:125–33. 10.1093/cvr/cvt178 23847387

[14] Egashira Y Suzuki Y Azuma Y Takagi T Mishiro K Sugitani S The growth factor progranulin attenuates neuronal injury induced by cerebral ischemia-reperfusion through the suppression of neutrophil recruitment. J Neuroinflammation (2013) 10:105. 10.1186/1742-2094-10-105 23972823 PMC3765381

[15] Yang D Wang LL Dong TT Shen YH Guo XS Liu CY Progranulin promotes colorectal cancer proliferation and angiogenesis through TNFR2/Akt and ERK signaling pathways. Am J Cancer Res (2015) 5:3085–97. 26693061 PMC4656732

[16] Krabbe G Minami SS Etchegaray JI Taneja P Djukic B Davalos D Microglial NFkappaB-TNFalpha hyperactivation induces obsessive-compulsive behavior in mouse models of progranulin-deficient frontotemporal dementia. Proc Natl Acad Sci U S A (2017) 114:5029–34. 10.1073/pnas.1700477114 28438992 PMC5441749

[17] Noguchi T Ebina K Hirao M Kawase R Ohama T Yamashita S Progranulin plays crucial roles in preserving bone mass by inhibiting TNF-alpha-induced osteoclastogenesis and promoting osteoblastic differentiation in mice. Biochem Biophys Res Commun (2015) 465:638–43. 10.1016/j.bbrc.2015.08.077 26297947

[18] Wang L Yang D Tian J Gao A Shen Y Ren X Tumor necrosis factor receptor 2/AKT and ERK signaling pathways contribute to the switch from fibroblasts to CAFs by progranulin in microenvironment of colorectal cancer. Oncotarget (2017) 8:26323–33. 10.18632/oncotarget.15461 28412748 PMC5432260

[19] Thurner L Fadle N Regitz E Kemele M Klemm P Zaks M The molecular basis for development of proinflammatory autoantibodies to progranulin. J Autoimmun (2015) 61:17–28. 10.1016/j.jaut.2015.05.002 26005049

[20] Etemadi N Webb A Bankovacki A Silke J Nachbur U Progranulin does not inhibit TNF and lymphotoxin-alpha signalling through TNF receptor 1. Immunol Cell Biol (2013) 91:661–4. 10.1038/icb.2013.53 24100384

[21] Chen X Chang J Deng Q Xu J Nguyen TA Martens LH Progranulin does not bind tumor necrosis factor (TNF) receptors and is not a direct regulator of TNF-dependent signaling or bioactivity in immune or neuronal cells. J Neurosci (2013) 33:9202–13. 10.1523/JNEUROSCI.5336-12.2013 23699531 PMC3707136

[22] Stubert J Waldmann K Dieterich M Richter DU Briese V Progranulin shows cytoprotective effects on trophoblast cells in vitro but does not antagonize TNF-alpha-induced apoptosis. Arch Gynecol Obstet (2014) 290:867–73. 10.1007/s00404-014-3296-3 25027814

[23] Jian J Zhao S Tian Q Gonzalez-Gugel E Mundra JJ Uddin SM Progranulin directly binds to the CRD2 and CRD3 of TNFR extracellular domains. FEBS Lett (2013) 587:3428–36. 10.1016/j.febslet.2013.09.024 24070898 PMC3826980

[24] Wang BC Liu H Talwar A Jian J. New discovery rarely runs smooth: an update on progranulin/TNFR interactions. Protein Cell (2015) 6:792–803. 10.1007/s13238-015-0213-x 26408020 PMC4624682

[25] Tsujimoto M Yip YK Vilcek J. Tumor necrosis factor: specific binding and internalization in sensitive and resistant cells. Proc Natl Acad Sci U S A (1985) 82:7626–30. 10.1073/pnas.82.22.7626 2999773 PMC391386

[26] Pichyangkul S Schick D Jia FL Berent S Bollon A Kahn A. Binding of tumor necrosis factor alpha (TNF-alpha) to high-affinity receptors on polymorphonuclear cells. Exp Hematol (1987) 15:1055–9. 2822458

[27] Niitsu Y Watanabe N Sone H Neda H Yamauchi N Maeda M Analysis of the TNF receptor on KYM cells by binding assay and affinity cross-linking. J Biol Response Mod (1988) 7:276–82. 2839624

[28] Pennica D Kohr WJ Fendly BM Shire SJ Raab HE Borchardt PE Characterization of a recombinant extracellular domain of the type 1 tumor necrosis factor receptor: evidence for tumor necrosis factor-alpha induced receptor aggregation. Biochemistry (1992) 31:1134–41. 10.1021/bi00119a023 1310420

[29] Pennica D Lam VT Mize NK Weber RF Lewis M Fendly BM Biochemical properties of the 75-kDa tumor necrosis factor receptor. Characterization of ligand binding, internalization, and receptor phosphorylation. J Biol Chem (1992) 267:21172–8. 1328224

[30] Pennica D Lam VT Weber RF Kohr WJ Basa LJ Spellman MW Biochemical characterization of the extracellular domain of the 75-kilodalton tumor necrosis factor receptor. Biochemistry (1993) 32:3131–8. 10.1021/bi00063a027 8384489

[31] Grell M Douni E Wajant H Löhden M Clauss M Maxeiner B The transmembrane form of tumor necrosis factor is the prime activating ligand of the 80 kDa tumor necrosis factor receptor. Cell (1995) 83:793–802. 10.1016/0092-8674(95)90192-2 8521496

[32] Lang I Füllsack S Wyzgol A Fick A Trebing J Arana JA Binding studies of TNF receptor superfamily (TNFRSF) receptors on intact cells. J Biol Chem (2016) 291:5022–37. 10.1074/jbc.M115.683946 26721880 PMC4777839

[33] Lang I Fick A Schäfer V Giner T Siegmund D Wajant H. Signaling active CD95 receptor molecules trigger co-translocation of inactive CD95 molecules into lipid rafts. J Biol Chem (2012) 287:24026–42. 10.1074/jbc.M111.328211 22645131 PMC3390677

[34] Fick A Lang I Schäfer V Seher A Trebing J Weisenberger D Studies of binding of tumor necrosis factor (TNF)-like weak inducer of apoptosis (TWEAK) to fibroblast growth factor inducible 14 (Fn14). J Biol Chem (2012) 287:484–95. 10.1074/jbc.M111.287656 22081603 PMC3249102

[35] Weiss T Grell M Hessabi B Bourteele S Müller G Scheurich P Enhancement of TNF receptor p60-mediated cytotoxicity by TNF receptor p80: requirement of the TNF receptor-associated factor-2 binding site. J Immunol (1997) 158:2398–404. 9036990

[36] Yamamoto Y Goto N Takemura M Yamasuge W Yabe K Takami T Association between increased serum GP88 (progranulin) concentrations and prognosis in patients with malignant lymphomas. Clin Chim Acta (2017) 473:139–46. 10.1016/j.cca.2017.07.024 28823651

[37] Yamamoto Y Takemura M Serrero G Hayashi J Yue B Tsuboi A Increased serum GP88 (progranulin) concentrations in rheumatoid arthritis. Inflammation (2014) 37:1806–13. 10.1007/s10753-014-9911-4 24803297

