# Supplementary material for: Lack of Evidence for a Direct Interaction of Progranulin and Tumor Necrosis Factor Receptor-1 and Tumor Necrosis Factor Receptor-2 From Cellular Binding Studies
Source: Front Immunol. 2018 Apr 23;9:793. doi: 10.3389/fimmu.2018.00793 (PMC5925078; doi:10.3389/fimmu.2018.00793)
Supplement: Supplementary file 1 [file Presentation_1.PDF]

SUPPLEMENTAL DATA – Lang et al

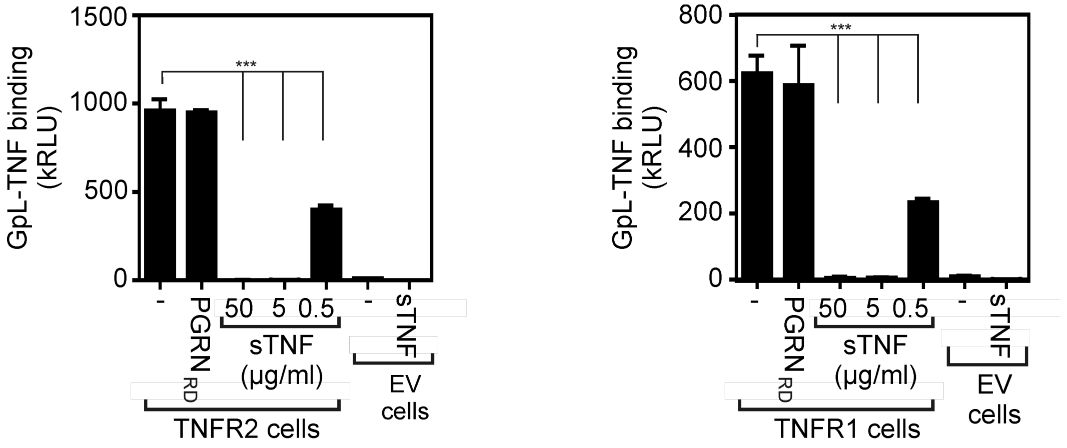

Supplementary Figure 1 I Preincubation with progranulin from R&D Systems (PGRN$_{RD}$) does not interfere with GpL-TNF binding to TNF receptors. HEK293 cells were transfected with empty vector (EV) or expression plasmids encoding TNFR1 or TNFR2. Next day, aliquots of cells (1 x 10^6) were preincubated with 500, 5.000 or 50000 ng/ml of sTNF or 50000 ng/ml PGRN$_{RD}$ for 1 h at 37°C or remained untreated. Binding studies were performed in technical triplicates with 20 ng/ml GpL-TNF. Please note, GpL-TNF binding of EV transfected cells in the presence and absence of an excess of sTNF defines the low endogenous expression of TNF receptors which was about 1 – 3 % of the ectopically expressed receptors.

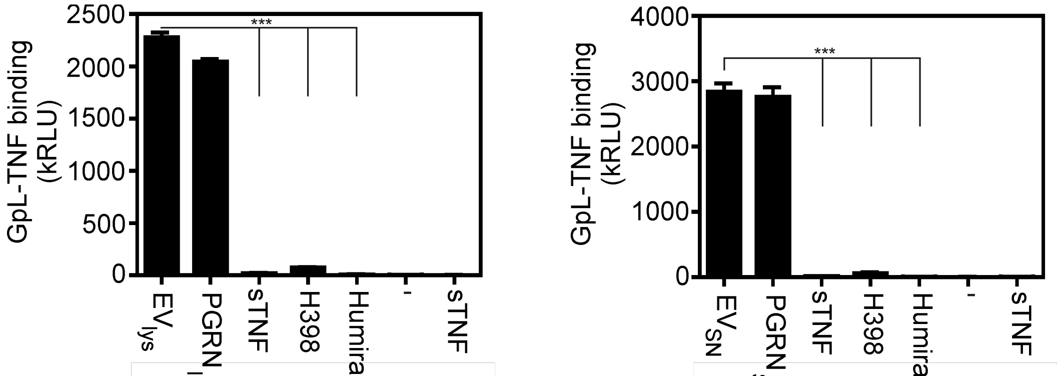

Supplementary Figure 2 I TNFR1-Fc or, as a control for unspecific binding, IgG1 was immobilized to black ELISA plates. Lysates (left panel) and supernatants (right panel) of progranulin and EV transfected cells (PGRN$_{lys}$ and EV$_{lys}$ (left panel), PGRN$_{SN}$ and EV$_{SN}$ (right panel)) were added for 1 h before the specific binding of 50 ng/ml GpL-TNF was determined in triplicates. PGRN concentrations of PGRN$_{lys}$ and PGRN$_{SN}$ were app. 15 µ/ml). Where indicated immobilized TNFR1-Fc was pretreated for 1 h with 2000 ng/ml sTNF or 20 µg/ml of a neutralizing TNFR1-specific antibody (H398). As an additional control GpL-TNF were pretreated for 1 h with 20 µg/ml of the TNF-neutralizing antibody (Humira).

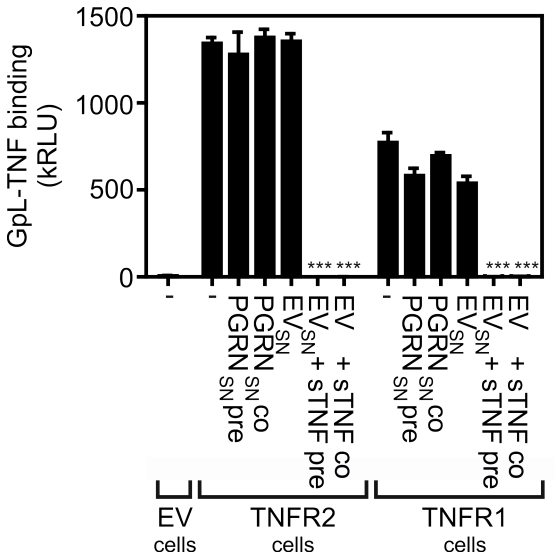

Supplementary Figure 3 I HEK293 cells were transfected with empty vector (EV) or expression plasmids encoding TNFR1 or TNFR2. Next day, aliquots of cells (1 x 10^6) were incubated for 1 h with 10 ng/ml GpL-TNF. Cells were furthermore pretreated (pre) for 30 min or cotreated (co) with $PGRN_{SN}$ (app. 15000 ng/ml), EV_{SN} or EV_{SN} supplemented with 20000 ng/ml of sTNF. Binding studies were performed in triplicates. $PGRN_{SN}$ and EV_{SN} = supernatants of HEK293 cells transfected with progranulin expression plasmid or empty vector.

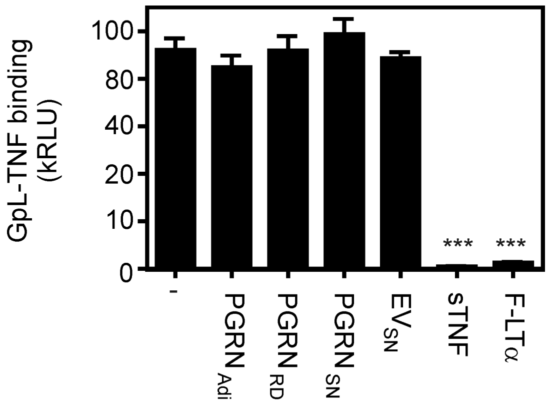

Supplementary Figure 4 I HeLa-TNFR2 cells (1 x 10^6) were incubated for 1 h with progranulin from Adipogen ($PGRN_{Adi}$, 25000 ng/ml), progranulin from R&D Systems ($PGRN_{RD}$, 25000 ng/ml), supernatant of HEK293 cells transfected with progranulin encoding and empty vector ($PGRN_{SN}$, app. 15000 ng/ml and EV_{SN}) and 2000 ng/ml of sTNF or Flag-LTα (F-LTα). Binding studies were then performed in triplicates with 50 ng/ml GpL-TNF for 1 h at 37°C.

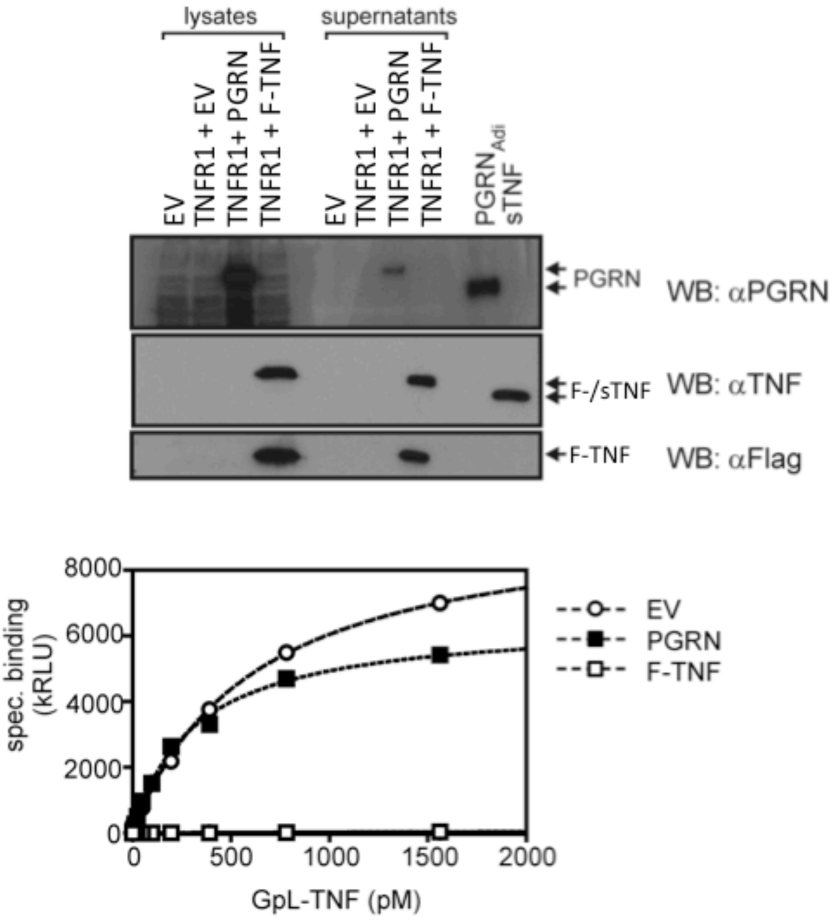

Supplementary Figure 5 I Upper panel: HEK293 cells were transfected with the indicated 1:1 mixtures of empty vector (EV) and death domain deletion mutant of TNFR1 (TNFR1), progranulin (PGRN) and Flag-TNF (F-TNF) encoding plasmids or with EV alone. The following day, lysates and supernatants of cells were were analyzed by Western blotting with anti-progranulin, anti-TNF and anti-Flag along with 100 ng $PGRN_{Adi}$ and 100 ng purified untagged soluble TNF (sTNF).

Lower panel: Equilibrium binding studies were performed with the indicated concentrations of GpL-TNF. Total GpL-TNF binding values from the progranulin (TNFR1 + PGRN transfection), Flag-TNF (TNFR1 + F-TNF transfection) and empty vector co-expressing TNFR1 (TNFR1 + EV tranfection) transfectants were subtracted from the corresponding unspecific binding values from cells only transfected with EV (transfection EV alone) to obtain specific binding. Specific binding values were fitted by non-linear regression analysis to a single binding site type of interaction by help of the GraphPad Prism 5 software. While there was > 99 % inhibition of specific GpL-TNF binding to the TNFR1 + Flag-TNF transfectants compared to the TNFR1 + EV transfectants, there was no significant change between the TNFR1 + EV and TNFR1 + PGRN transfectants.

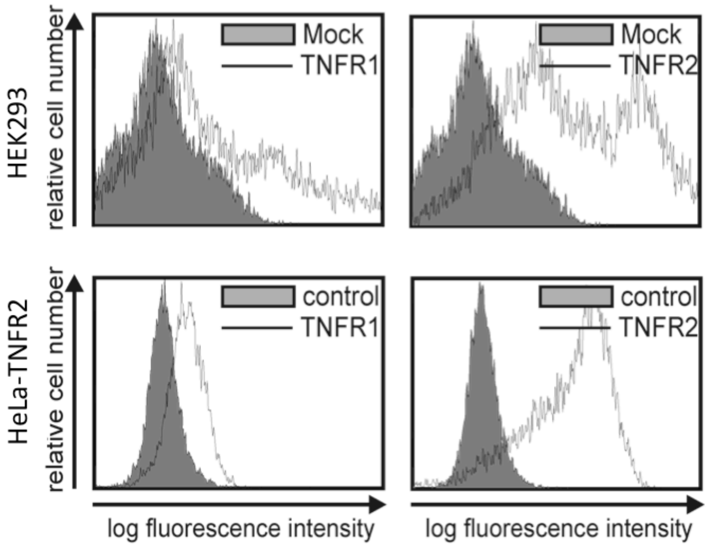

Supplementary Figure 6 I Upper panel: HEK293 cells were transfected with empty vector (mock) or expression plasmids encoding a deletion mutant of TNFR1 where the death domain has been replaced by YFP (TNFR1) or a deletion mutant of TNFR2 where the TRAF2 binding site has been replaced again by YFP (TNFR2). Next day, cells were analyzed by FACS. Lower Panel: HeLa-TNFR2 cells (here alternatively named HeLa80) were analyzed by FACS with respect to TNFR1 and TNFR2 expression using TNFR1- and TNFR2-specific antibodies or a corresponding IgG control antibody.

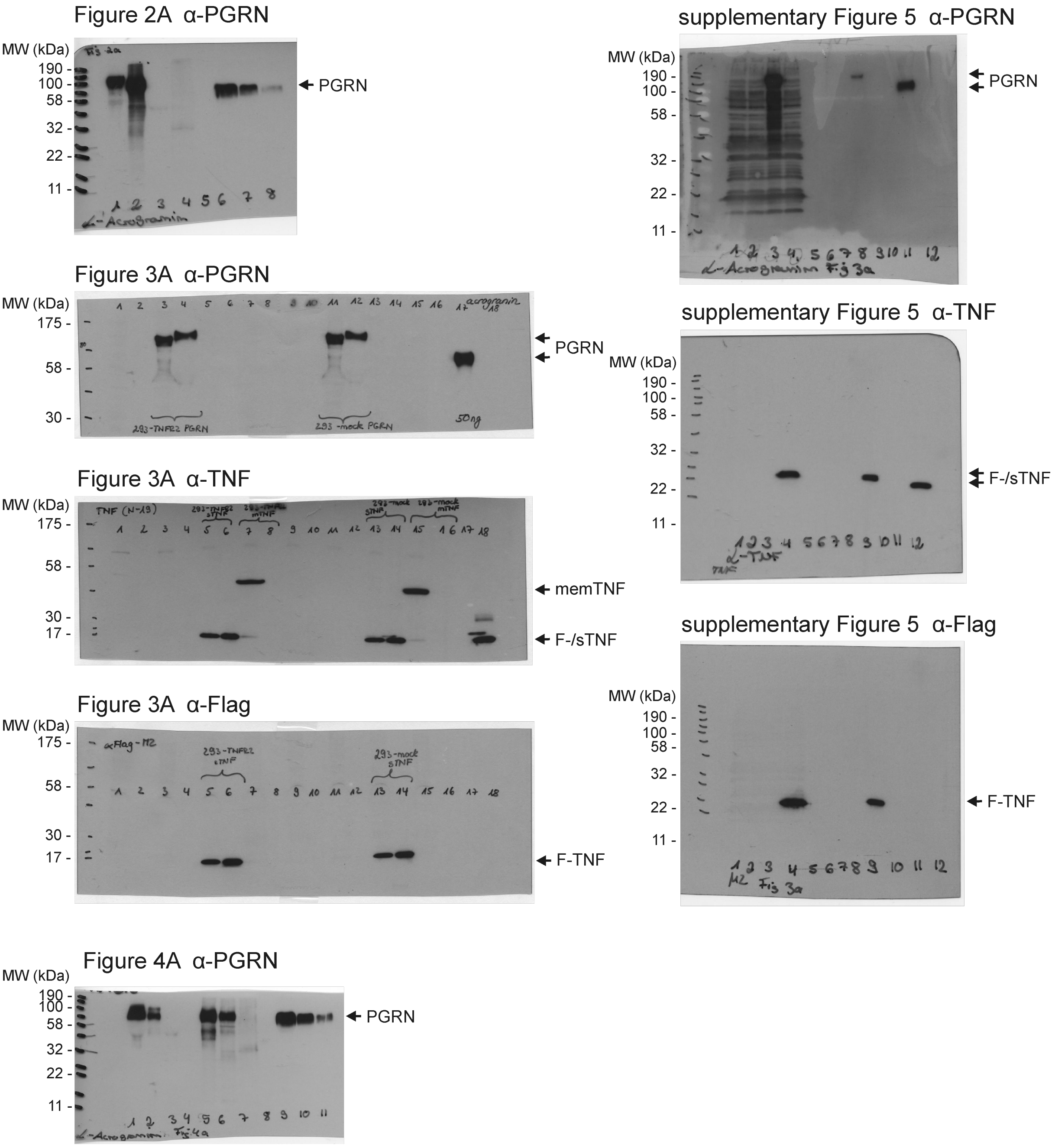

Supplementary Figure 7 I Complete Western blots of the cuttings shown in Figures 2A, 3A and 4A. Antibodies used are indicated. The position of prestained marker proteins on the nitrocellulose membrane were transferred to the X-ray film by hand with felt pen. As marker proteins the Blue Prestained Standard, Broad Range (7-175 kDa) mixture (not anymore available) and Blue Prestained Standard, Broad Range (11-190 kDa) mixture from New England Biolabs have been used. For lack of space only every second marker position were labelled with its size. In the case of the Blue Prestained Standard, Broad Range (7-175 kDa) the 7 kDa marker had been run out from the gel.
